# Incidence, risk factors, and outcomes of unplanned extubation in adult patients in a resource-limited teaching hospital in the Philippines: a cohort study

**DOI:** 10.5935/0103-507X.20190012

**Published:** 2019

**Authors:** Angelique Bea C Uy, Everly Faith P Ramos, Adovich S Rivera, Norman L Maghuyop, Cezar Thomas R Suratos, Red Thaddeus D Miguel, Mairre James S Gaddi, Joan Kristi D Zaldivar

**Affiliations:** 1 Department of Medicine, Philippine General Hospital, University of the Philippines -Manila, Philippines.; 2 Institute of Health Policy and Development Studies, National Institutes of Health, University of the Philippines - Manila, Philippines.; 3 Department of Neurosciences, Philippine General Hospital, University of the Philippines - Manila, Philippines.; 4 Department of Surgery, Philippine General Hospital, University of the Philippines - Manila, Philippines.

**Keywords:** Intubation, Airway extubation, Critical care, Developing country, Prospective studies

## Abstract

**Objective:**

We aimed to determine the incidence, risk factors, and outcomes of unplanned extubation among adult patients.

**Methods:**

We conducted a prospective cohort study of adult intubated patients admitted to the charity wards of a government tertiary teaching hospital in the Philippines. Patients managed in both intensive care and nonintensive care settings were included. Patients were followed-up until discharge or until seven days postextubation.

**Results:**

The outcomes of the 191 included patients were planned extubation (35%), unplanned extubation (19%), death (39%), and discharge against advice (7%). Competing risk regression showed that male sex (Crude OR: 2.25, 95%CI: 1.10 - 4.63) and age (Crude OR 0.976, 95%CI: 0.957 - 0.996) were significant baseline factors. The night shift (Crude OR: 24.6, 95%CI: 2.87 - 211) was also consistently associated with more unplanned extubations. Among postextubation outcomes, reintubation (unplanned extubation: 61.1% *versus* planned extubation: 25.4%), acute respiratory failure (unplanned extubation: 38.9% *versus* planned extubation: 17.5%), and cardiovascular events (unplanned extubation: 8.33% *versus* planned extubation: 1.49%) occurred significantly more often among the unplanned extubation patients. Admission in an intensive care unit was not associated with a lower risk of unplanned extubation (Crude OR 1.15, 95%CI: 0.594 - 2.21).

**Conclusion:**

Many intubated patients had unplanned extubation. Patients admitted in nonintensive care unit settings did not have significantly higher odds of unplanned extubation.

## INTRODUCTION

Unplanned extubation (UE) is the "premature removal of the endotracheal tube by action of the mechanically ventilated patient (self-extubation) or premature removal during nursing and medical care (accidental extubation)".^(^^1^^)^ It is considered the most common adverse event occurring in the intensive care unit (ICU)^(^^2^^)^ and was estimated to occur in 7% to 22.5% of intubated patients.^(^^3^^)^ More recent studies cite a lower incidence of UE, such as a center in Korea reporting UE occurring at a rate of 0.6%.^(^^4^^)^ Unplanned extubation is considered to be an indicator of quality of care for critically ill patients^(^^5^^)^ as it is associated with increased morbidity, mortality and cost in a majority of studies on UE.^(^^6-8^^)^ However, there could be variations between centers, as some reported not having an increased mortality risk.^(^^1,9^^)^

Several reviews have been published related to UE and its risk factors.^(^^8,10,11^^)^ Although the majority of the studies included in these reviews have been conducted in high-resource countries, the gap in knowledge is slowly being addressed by recent publications that investigate UE in developing countries.^(^^12-16^^)^ In these studies conducted in low-middle income countries, the proportion of intubated patients who experienced UE ranged from 0.1% to 10.3%, which is comparable to high-resource countries.

Hospitals in developing countries are not always able to provide care in ideal settings. Due to limited ICU capacities, some intubated patients may be admitted in the general wards. The published studies we found, however, have not been able to include this in their discussions.

We aim to address this gap in knowledge and relay the experience of caring for intubated patients in a resource-limited setting. In this study, we measured the incidence of UE under the charity service of a tertiary government hospital in the Philippines. We also compared the short-term postextubation outcomes of patients who experienced UE to the outcomes of those who underwent planned extubation (PE).

## METHODS

This observational prospective cohort study was conducted at the Philippine General Hospital (PGH) from February to May 2017. With a capacity of 1500 beds, PGH is the largest government teaching hospital in the Philippines. All charity wards and ICUs, except for two wards, were included. Nonsurgical wards were the Internal Medicine (2 wards, 1 ICU) and Neurosciences (1 ward, 1 ICU) wards. Surgical wards were the Surgery (3 wards, 1 ICU), Neurosciences (1 ward, 1 ICU), and Obstetrics-Gynecology (3 wards, 1 ICU) wards. Excluded from the study were the Ophthalmology ward and the special ward for government-insured patients because these two wards do not handle intubated patients. General wards have 1:13 nurse to patient ratios, while ICUs have 1:4.

All intubated patients hooked to mechanical ventilators, 19 years or older, and admitted in any of the selected wards or ICUs were included in the study. Patients who were intubated for surgery and were extubated at the operating room were excluded. Patients who were intubated at the Emergency Department and discharged without being admitted to the ward or ICU were also excluded from the study. Finally, intubated patients at the start of recruitment who had a previous history of UE during the same admission were not included since we were interested in the first UE event only, and we would be unable to collect postextubation outcomes on these patients.

The team had a target of at least 103 patients to estimate a UE rate of 22%^(^^3^^)^ with an 8% margin of error and an alpha of 0.05.

The main outcome was UE. Competing outcomes were PE, death prior to extubation, and discharge against advice (DAA) prior to extubation. Postextubation outcomes were reintubation, acute respiratory failure, cardiovascular events, nosocomial infection, and death. Chart diagnoses were used as the basis for postextubation events.

There were two types of risk factors considered based on previous literature: baseline and circumstance. Baseline factors included age, sex, type of ward (surgical *versus* nonsurgical), ICU admission, reason for intubation, initial Simplified Acute Physiology Score (SAPS) II, and ventilation mode (continuous positive airway pressure - CPAP - *versus* other ventilation modes such as assisted-control ventilation). Baseline factors were collected for all patients. The reason for intubation was based on diagnoses and problem lists in the charts. For the SAPS II score, none of the patients had bilirubin results, and the scores were computed without this component.

Circumstantial factors were timing of extubation, use of restraints, use of sedation 24 hours prior to extubation, type of fixation used, and use of X-ray confirmation for tube placement. These were collected only for those who had PE or UE.

Upon inclusion in the study, research staff completed data collection forms by extracting demographic and clinical data from charts. Residents and nurses in charge of the patient were interviewed if there were details missing in the chart. Daily visits were performed for outcome assessment. If UE or PE occurred, data on circumstantial factors were collected. The extubated patients were then tracked up to seven days postextubation or up to discharge, whichever was shorter, for postextubation outcomes.

### Data analysis

Outcome incidence was computed by dividing the number of UEs over the cumulative person-days of observation. Three-day incidence densities from intubation day to the 15^th^ day of intubation were computed to assess trends.

Patients were classified according to multiple outcomes and described using demographic and clinical variables. Categorical variables were compared using the Chi-square test. Age and SAPS II score were compared using one-way ANOVA, while length of stay and intubation were compared using the Kruskal-Wallis rank test due to nonnormality of data.

Due to the presence of multiple outcomes, competing risk regression was used to estimate subhazard ratios (SHRs) for the baseline factors. Due to the small group size, DAA was grouped with death. Logistic regression was used to test for the association of circumstantial factors with UE, controlling for baseline variables. Collinearity was assessed using the "Collin" command.

We planned to run analysis including all risk factors (full model) but due to concerns of the small sample size, limited number of events, and overfitting, we also ran backwards stepwise models (at a threshold of p ≤ 0.20) to reduce the number of terms in the model. As a sensitivity analysis, we also ran models where the SAPS II score was excluded to allow inclusion for all patients in the cohort.

The failure rate, defined as reintubation within 48 hours of extubation, was computed. Kaplan-Meier failure curves of the postextubation outcomes were plotted for UE and PE patients. These curves were compared using the Gehan-Breslow-Wilcoxon method due to the variation of incidence rates over the follow-up period. No adjusted analyses were performed due to the small sample size.

A p value less than or equal to 0.05 was considered significant. All statistics were calculated using Stata 12 software.

This study was approved by the Technical Review Board and Ethics Review Board of the University of the Philippines, in Manila.

## RESULTS

The study registered a total of 195 intubated patients. Four patients were excluded due to incomplete outcome data, thus only 191 patients were included in the analysis. The mean age of enrolled patients was 54 (standard deviation - SD: 15.5) years old, with a slight dominance of the male population (55.5%). The patients were roughly equally distributed between the ICU (53.9%) and the non-ICU setting (46.1%). There were slightly more enrolled patients admitted under the nonsurgical wards (73.3%) compared to surgical ones and under the ICU (55%) wards compared to non-ICU wards. Only 32 (16.41%) were admitted for surgical reasons. The average and median length of stay in days were 23.75 (SD: 25.16) and 17 [interquartile range - IQR: 22], respectively. The average SAPS II score was 49 (n = 143, SD: 0.26) (Table 1).

**Table 1:** Patient characteristics according to outcome

Patient characteristics	Whole cohort n = 191	Unplanned extubation n = 36	Planned extubation n = 67	Death or DAA n = 88	p value
Age	54.8 (15.5)	49.7 (15.8)	56.4 (14.6)	55.5 (16.0)	0.091*
Female	44.5	27.8	53.7	47.3	0.044†
Mean SAPS II‡	49.0 (0.26)	50.4 (13.9)	46.1 (14.5)	52.2 (13.5)	0.151*
Admission to the ICU	55.0	58.3	35.1	38.7	0.032
Admission to nonsurgical area	73.3	58.3	67.2	45.9	0.063†
Reasons for intubation					
Cardiovascular	24.1	19.4	29.9	23.0	0.548†
Pulmonary	43.5	33.3	35.6	54.1	0.081†
Sepsis	12.0	11.1	7.5	14.9	0.331†
Neurologic	37.2	47.2	40.3	33.8	0.146†
Surgical and others	7.9	2.8	9.0	8.1	0.752†
CPAP mode (*versus* other MV modes)	14.1	19.4	7.5	16.2	0.180†
Median length of stay (days)	17 [22]	21.5 [23.5]	26 [28]	10 [16]	< 0.001§
Median length of intubation (days)	8 [10]	4.5 [7.5]	8 [9]	10 [16]	< 0.001§

DAA - discharge against advice; SAPS II - simplified acute physiology score II; ICU - intensive care unit; CPAP - continuous positive airway pressure; MV - mechanical ventilation.

* One-way ANOVA;

† Chi-square test;

‡ n are as follows: whole - 143; unplanned extubation - 30; planned extubation - 53; death/discharge against advice - 60;

§ Kruskal-Wallis rank test.

Results expressed by mean (standard deviation), % or median [interquartile range].

### Unplanned extubation and competing outcomes

Of the entire cohort (n = 191), there were 67 (35%) PE and 36 (19%) UE patients. Seventy-four patients (39%) died before extubation, while 14 patients (7%) were DAA. If we count only patients who were alive on discharge and not DAA in the denominator, 35.0% (n = 103) had UE. Of the 36 UE patients, the majority (28, 77.8%) of UE was due to self-extubation. The average and median length of intubation in days were 12.21 (SD: 10.82) and 8 [IQR: 10], respectively.

The incidence rate of UE was 1.55 per 100 person-days (95%CI: 1.11 - 2.14), while that of PE was 2.83 per 100 person-days (95%CI: 2.23 - 3.61). Patients admitted in an ICU setting had a slightly higher incidence of UE (1.68 per 100 person-days, 95%CI: 1.10 - 2.58) compared to those in non-ICU settings (1.39 per 100 person-days, 95%CI: 0.83 - 2.30).

The incidence of death and DAA were 3.18 per 100 person-days (95% CI: 2.53 - 3.99) and 0.6 per 100 person-days (95%CI: 0.36 - 1.01), respectively. The incidence density of UEs and PEs peaked at the 3^rd^ to 6^th^ intubation day interval. Death peaked at the 6^th^ to 9^th^ day interval, while DAA peaked at the 9^th^ to 12^th^ day interval.

### Risk factors for unplanned extubations

For baseline risk factors, only age (0.976, 95%CI: 0.957 - 0.996) and male sex (2.25; 95% CI: 1.10 - 4.63) had significant crude subhazard ratios for UE. Male sex remained significant after adjustment using the full model (2.54, 95%CI: 1.09 - 5.94) and the stepwise model (2.39, 95%CI: 1.09 - 5.27). Age was significant in the full model (0.969, 95%CI: 0.941 - 0.999) but not in the stepwise model (0.975; 95%CI: 0.950 1.00). Admission in an ICU setting was not a significant factor for UE in either the crude (1.15, 95%CI: 0.594 - 2.21) or adjusted analysis (1.04, 95%CI: 0.481 - 2.23). The associations found were consistent in the sensitivity analysis, although age remained significant in the stepwise model (Table 2).

**Table 2:** Subhazard ratios of baseline characteristics for unplanned extubation

Patient characteristics	Crude SHR	Adjusted SHR (full model)	Adjusted SHR (stepwise)
Age	0.976* (0.957 - 0.996)	0.969* (0.941 - 0.999)	0.975 (0.950 - 1.00)
Male sex (reference: female)	2.25* (1.10 - 4.63)	2.54* (1.09 - 5.94)	2.39* (1.09 - 5.27)
Admission to the ICU	1.15 (0.594 - 2.21)	1.04 (0.481 - 2.23)	Not included
Admission to nonsurgical area	0.711 (0.360 - 1.41)	0.473 (0.168 - 1.33)	Not included
Primary diagnosis for intubation			
Cardiovascular	0.762 (0.332 - 1.75)	1.13 (0.389 - 3.29)	Not included
Pulmonary	0.616 (0.311 - 1.22)	0.512 (0.190 - 1.38)	0.441 (0.184 - 1.06)
Sepsis	0.959 (0.327 - 2.812)	1.29 (0.313 - 5.317)	Not included
Neurologic	1.53 (0.802 - 2.92)	0.988 (0.404 - 2.42)	Not included
Surgical and others	0.669 (0.162 - 2.77)	0.162 (0.010 - 2.51)	0.221 (0.265 - 1.84)
CPAP mode (*versus* other MV modes) (%)	1.50 (0.672 - 3.35)	1.79 (0.503 - 6.37)	Not included
SAPS II	1.01 (0.981 - 1.03)	1.00 (0.974 - 1.03)	Not included

SHR - standardized hazards ratio; ICU - intensive care unit; CPAP - continuous positive airway pressure; MV - mechanical ventilation; SAPS II - simplified acute physiology score II.

* p value < 0.05. Results expressed by standardized hazards ratio point estimate and 95% confidence interval (in parentheses).

For circumstantial factors, the night shift was associated with increased odds of UE (24.6, 95%CI: 2.87 - 211), and significance remained after adjustment. Sedation was found to be associated with decreased odds of UE (0.052, 95%CI: 0.004 - 0.777), while the use of physical restraints was associated with increased odds in adjusted analysis using the full model (8.439, 95%CI: 1.40 - 51.0). These factors were not significant using the stepwise model or in the model where the SAP II score was not included in the covariates (Table 3).

**Table 3:** Odds ratios of circumstantial factors for unplanned extubation

Circumstantial factors	Unplanned extubation (%)	Planned extubation (%)	Odds ratio (95% confidence interval)		
			Crude	Adjusted (full model)*	Adjusted (stepwise)†
Timing					
AM	41.7	63.1	-	-	-
PM	33.3	35.4	1.42 (0.571 - 3.56)	2.76 (0.449 - 17.0)	2.31 (0.73 - 7.32)
Night	25.0	1.5	24.6‡ (2.87 - 211)	86.3‡ (2.44 - 3,050)	37.0‡ (3.10 - 442)
Use of restraints	41.7	29.9	0.518 (0.722 - 3.90)	8.439‡ (1.40 - 51.0)	2.88 (0.939 - 8.84)
Use of sedation	5.6	26.9	0.160 (0.035 - 0.736)	0.053‡ (0.004 - 0.777)	0.284 (0.052 - 1.56)
Tape for fixation	11.1	3.0	1.40 (0.706 - 23.4)	Omitted (predicts success perfectly)	Not included
X-ray confirmation	91.7	85.1	0.657 (0.496 - 7.52)	1.00 (0.050 - 20.0)	Not included

AM - ante meridiem; PM - post meridiem.

* Models included baseline risk factors

† Final model included age, sex, surgical and other reasons for intubation, mechanical ventilation mode, admission in nonsurgical wards.

‡ p-value <0.05.

We explored whether there were differences in the odds of UE during the night shift stratified according to ICU admission and found no significant difference between the two ORs (Chi-square p-value = 0.49). We also explored whether there were differences in the OR of UE for sedation according to the mode of mechanical ventilation (CPAP *versus* not CPAP) and found no significant difference (Chi-square p-value = 0.08). There were not enough events and samples to test for interactions.

### Postextubation outcomes

The overall failure rate was 34.0%, with a lower rate for PE (20.9%) compared to UE (58.3%). This translated to a lower odds ratio of failure if the extubation was planned (Crude OR 0.187, 95%CI: 0.078 - 0.458, p-value < 0.001).

Reintubation (UE: 61.1% *versus* PE: 23.9%), acute respiratory failure (UE: 38.9% *versus* PE: 16.4%), and cardiovascular events (UE: 8.33% *versus* PE: 1.49%) occurred more often in the UE group. Failure curves for these three outcomes were significantly different (p-values < 0.05). There was no significant difference in the occurrence of infection (UE: 27.8% *versus* PE: 22.4%) or death (UE: 22.2% *versus* PE: 11.9%) between the UE and PE patients (Table 4).

**Table 4:** Occurrence of postextubation outcomes according to type of extubation

Postextubation outcome	Unplanned extubation (n = 36)	Planned extubation (n = 67)	Wilcoxon-Breslow test* p value
Reintubation	22 (61.1)	16 (23.9)	< 0.001
Acute respiratory failure	14 (38.9)	11 (16.42)	0.002
Cardiovascular event	3 (8.33)	1 (1.49)	0.045
Infection	10 (27.8)	15 (22.4)	0.273
Death	8 (22.2)	8 (11.9)	0.112

* Comparison of Kaplan-Meier failure curves. Results expressed by count and percent (in parentheses).

## DISCUSSION

In this single center cohort study, we found that approximately 19% of intubated patients admitted under the charity service experienced UE with an incidence density of 1.55 per 100 person-days. This is consistent with the published rates of 7 - 22%,^(^^3^^)^ or 0.1 to 3.6 per 100 intubation-days.^(^^10^^)^ If deaths and DAA were removed in the equation, the occurrence in this setting becomes much higher at 35%. This higher incidence rate would exceed the highest UE rate reported from a low- and middle-income country.^(^^12^^)^

We did not expect UE rates between ICU and non-ICU patients to be similar. We hypothesized that ICU patients would have lower rates because ICU patients would have more severe diseases, would be weaker, and would be less likely to self-extubate. Another consideration is that the ICU setting is expected to have a greater capacity to provide ideal care. We addressed the first concern by adjusting for severity using the SAPS II in the multivariable models and found that it was not a significant predictor of UE. Although we do not have evidence related to quality of care in the ICU or wards, it may be that the wards must have created an environment that approximated ICU care. In this center, intubated general ward patients are assigned to special detail beds. These patients are attached to the same monitoring machines as ICU patients and are visited frequently by medical clerks and interns. These adaptations might have been enough to address care issues such as the suboptimal nurse to patient ratio. It could also be that residents unintentionally assign patients who are less at-risk for UE to the general ward instead of the ICU.

Our findings about baseline and circumstantial factors were not always consistent with the findings of a comprehensive review by da Silva et al.^(^^10^^)^ on this topic. Age and sex were significant risk factors in our study; however, age was cited as a significant risk factor in only three of 11 studies, and sex was significant in only one of three studies reviewed. On the other hand, our study is consistent with most studies included in the review citing physical restraint as a risk factor for UE. Two studies in the same review also found night time to be a risk factor for UE. Our study, however, failed to detect significant consistent protection conferred by the type of tape used to secure the endotracheal tube, unlike the studies that investigated it. We did not find a significant increase or decrease in the odds of UE with sedative use, unlike the studies cited in the review.

Our study agreed with a previously observed increase in the need for reintubation among UE patients,^(^^1^^)^ but did not confirm findings of increased death or infection observed in other studies.^(^^7,8^^)^ This failure to detect differences may be attributed to the low sample size and events of our study.

This is one of the few studies that looked at UE in a resource-limited hospital and, to the best of our knowledge, is the only study that included intubated patients managed in a non-ICU setting. Another strength of this study is the use of competing risk regression instead of logistic regression to account for the presence of more than one common alternative outcome.

There is a limitation of generalizability as this was conducted in a single center in the Philippines. In addition, we were not able to include patients who were managed and discharged at the Emergency Department. If they were included, our incidence would likely have been higher.

The relatively small sample size and number of events led to lower power to detect significant associations. Due to concerns of overfitting related to the small sample size and number of events, we conducted sensitivity analysis and found that most of the results were robust. Our findings, however, should not be used to conclude that other previously published UE risk factors and outcomes are not significant for patients in this setting. A study with a higher sample size would be able to overcome these limitations. Nevertheless, this study could serve as baseline data for future quality improvement programs to be implemented in this hospital.

## CONCLUSION

Patients admitted in the charity wards and intensive care units experienced unplanned extubation. There was no detected difference in rates between patients admitted in an intensive care unit *versus* those admitted in a ward. Baseline clinical characteristics associated with unplanned extubation were male sex and younger age. The night shift was associated with an increased risk of unplanned extubation. Unplanned extubation increased the risk for reintubation, acute respiratory failure, and cardiovascular events, but no increased risks in postextubation death or infection were detected.
